# Electronic spectra of ions of astrochemical interest: from fast overview spectra to high resolution†

**DOI:** 10.1039/c8fd00196k

**Published:** 2018-12-12

**Authors:** Jana Roithová, Juraj Jašík, Jesus J. Del Pozo Mellado, Dieter Gerlich

**Affiliations:** Institute for Molecules and Materials, Radboud University Heyendaalseweg 135 6525 AJ Nijmegen Netherlands jana.roithova@ru.nl; Department of Organic Chemistry, Faculty of Science, Charles University Hlavova 2030/8 128 43 Prague 2 Czech Republic; Department of Physics, University of Technology 09107 Chemnitz Germany

## Abstract

The combination of cryogenic ion traps with suitable light sources and standard tools of mass spectrometry has led to many innovative applications in previous years. This paper presents the combination of our versatile instrument with a supercontinuum laser for the rapid identification of ions that might be of special interest, *e.g.* as candidates for diffuse interstellar bands carriers. Using a linear wire quadrupole ion trap at 3 K, routine He-tagging, long irradiation times, and the brilliance and wide spectral range of a crystal fiber laser, mass selected ions have been exposed to spectral fluencies larger than 10 mJ (nm cm^2^)^−1^. These conditions result in an unsurpassed sensitivity, allowing us to find out within a few minutes and with nm accuracy, where photo absorption occurs with cross sections above 10^−18^ cm^2^. In this contribution, we present a variety of ions, probed between 420 and 720 nm. They have been generated by electron- or electrospray ionization of (polycyclic) aromatic hydrocarbons. For selected candidates, we recorded spectra with higher resolution and in the IR range. The anthracene dication has been selected to present a detailed analysis of our new results.

## Abstract 2

Combining He-tagging in a cryotrap with a supercontinuum laser is an efficient way of identifying candidates for DIBs carriers.

## Graphical abstract

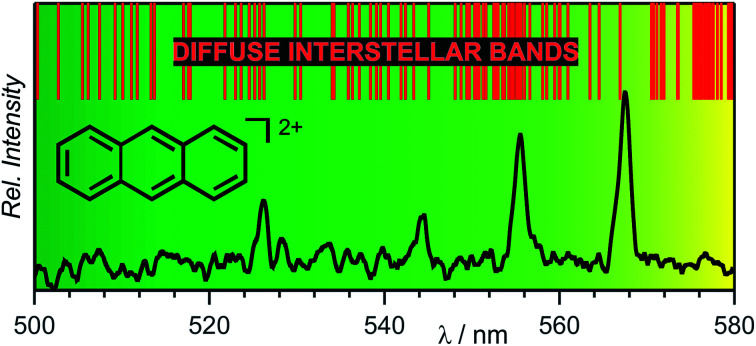

## 1. Introduction

Diffuse interstellar bands (DIBs) are one of the puzzles of astrochemistry that has persisted for almost 100 years.^1^ While it is known that DIBs are spectral signatures of molecules/ions present in interstellar medium, it is not known what exactly these molecules/ions are.^2^ Based on boundary conditions, astronomers expect that most of the DIBs precursors originate from a family of hydrocarbon and/or polycarbon molecules.^3–7^ The lure of assigning the DIBs to concrete molecules/ions has attracted many laboratory spectroscopists.^8–12^ However, given the amount of possible DIBs precursors, the probability of a positive hit is exceedingly small.^13^

Despite the unfavourable win/loss ratio, the group of John P. Maier succeeded in assigning two DIBs to the C_60_^+^ cation in 2015.^14^ The work was based on earlier predictions suggesting that the C_60_^+^ cation is one of the DIBs carriers. It took more than 20 years to perform convincing laboratory experiments.^15,16^ Today, we remain with about 400 DIBs to be assigned in future. Clearly, if one intends to succeed with assigning more DIBs, one needs more efficient strategies to screen the spectra of possible precursors than those we have used until today. Here, we present an efficient way to screen the spectra of ions in the gas phase using the helium tagging method together with a high brilliance supercontinuum laser. We demonstrate this procedure for selected PAH (polycyclic aromatic hydrocarbon) cations.

### Major laboratory spectroscopic tools in search of DIBs precursors

In order to bring our methodology in line with other existing approaches, we give a short overview of activities devoted to the investigation of species of astrochemical interest. The final aim of such experiments must be to record unperturbed gas phase spectra under the conditions prevailing in space, *i.e.* at low densities and low temperatures.

Many results, especially for PAH molecules, originate from matrix-isolation spectroscopy.^17^ This well-established technique confines molecules, radicals or mass selected ions in an inert medium at very low temperatures. It is useful for obtaining an approximate overview of band positions; however, it has the disadvantage that, due to the interaction of the molecules with the rare gas, the positions and shapes of the absorption bands are perturbed.^18^ Also, para-hydrogen can serve as a host in matrix-isolation experiments.^19^ The influence of this matrix on the spectral features is reported to be smaller than that of noble-gas matrices. A closely related method is based on incorporating neutral or charged molecules into helium nanodroplets.^20,21^ The liquid matrix provides a constant temperature of 0.4 K. The spectra are recorded mainly using mass-specific depletion techniques and are closer to that of the gas phase due to the significantly reduced interaction with the host.

A very versatile method for studying gas phase molecules in a cold and interaction-free environment is the molecular beam method.^22^ High-pressure rare gas enriched with a few % of the studied molecules is expanded through a small nozzle into a vacuum and transformed to a freely propagating molecular beam. The low densities require very sensitive detection methods, such as laser-induced fluorescence, resonance-enhanced multiphoton ionization or multiple photon dissociation. Due to competing processes, extraction of the absorption spectra is not straightforward.

The direct absorption of light in supersonic expansions has been detected successfully using cavity ring-down spectroscopy.^23^ This method determines the rate (rather than the magnitude) of absorption through monitoring the decay of laser intensity in a high quality resonator. Also, many absorption spectra of ions have been measured by attaching a tag in a supersonic expansion, followed by photodissociation.^24–26^

Ion traps are suitable tools to reproduce the conditions of space in laboratory experiments.^27,28^ The visible photodissociation spectra of PAH cations and their derivatives have been measured in the PIRENEA experiment^29^ or in cryogenic Paul traps.^30^ Also, the cryogenic storage ring, CSR, aims to study molecules under interstellar conditions. It has been used recently to study the photofragmentation of cold CH^+^ using UV light.^31^ Different applications of cold ion spectroscopy have been presented in ref. 32–36. A recent review^37^ describes the long path from the idea to use He as a tag for spectroscopy to recent achievements, allowing us now to routinely attach He to mass selected ions inside a trap.^38–40^*In situ* synthesis also made it possible to record the gas phase electronic spectra of cold C_60_^+^ and to confirm that at least two diffuse interstellar bands (DIBs) are due to this ion.^14,41^

In recent years, dedicated cryogenic ion trap instruments have started to contribute to the search for the carriers of the DIBS;^14,42,43^ however, there are still discussions about the “optimum” arrangement.^44^ In the following, we demonstrate how one can optimize the interplay between suitable lasers and a linear quadrupole trap. This together with He-tagging significantly increases the efficiency of searching for the carriers of DIBs or for other spectra of interesting molecules.

## 2. Experimental and computational details

Ion spectroscopy experiments were performed using the versatile instrument ISORI described in detail elsewhere (see also Fig. S1 in the ESI†).^45,46^ It combines a cryogenic linear quadrupole ion trap (w4PT) with modules from a commercial TSQ 7000 instrument. A large variety of ions can be generated using the commercially available ion sources. In the present study, most ions were generated using electron ionization of neutral precursors heated in the solid probe. In one case, we also used electrospray ionization. The ions of interest were mass selected using a quadrupole (the resolution of the quadrupole was adjusted to mass-select ions with only one given *m*/*z* ratio) and transferred *via* a quadrupole bender and an octopole to the ion trap operated at 3 K, where they were cooled using 1 to 3 short helium pulses. Already during this process, a sufficient number of ions attach one helium atom (typically a few thousand). After 980 ms, all ions were extracted and mass analyzed using a second quadrupole. The RF is switched off for 5 ms. In total, one measurement cycle has a duration of 1 second. Besides the primaries and the singly tagged ions, there are a few ions with two He atoms and traces of ions tagged with H_2_O or N_2_ (switching reaction with gas impurities).

### Recording and presenting spectra

The ion cloud is confined by the effective potential close to the axis of the linear quadrupole (see Fig. S2 in the ESI†). The cloud is irradiated with photons in the IR, vis or UV range. The overall fluence the ions are exposed to is controlled by changing the power or the energy per pulse of the light source, by changing the number of pulses/irradiation time (the irradiation time was typically varied between 100 and 850 ms) or by changing the geometry of the light beam (see below). For recording spectra, we set the second quadrupole to the mass of the complexes with one He and counted these ions with and without light, resulting in numbers *N*(*ν*) and *N*_0_, respectively. Spectra are presented by plotting the attenuation of He-complexes, 1 − *N*(*ν*)/*N*_0_, as a function of the frequency. For many ions, our experiment can be operated such that the signal is strongly saturated and that non-linear optical effects play a role. Attenuations of 1 can be reached if there are no isomers or other ions in the trap which do not absorb. Absolute cross sections can be determined directly from the measured attenuation (see eqn (S6)†) or, more precisely, by measuring the attenuation as a function of the fluence. For a detailed description of the method and a discussion of its accuracy and experimental problems see the ESI.†

### Light sources

IR spectra are measured using a pulsed OPO/OPA system (LaserVision, 10 Hz repetition rate, resolution ∼1.6 cm^−1^ FWHM, pulse length 10 ns). The overall tuning range is 600–7400 cm^−1^ (Signal: 4700–7400 cm^−1^; Idler: 2000–4700 cm^−1^; with extension crystal: 600–2100 cm^−1^). The OPO is pumped by a seeded Nd:YAG laser (Surelite EX from Continuum). During acquisition of the spectra, the energy of each photon pulse is recorded using a laser energy meter Coherent Fieldmax II with a J-25MB-LE sensor.

For probing molecular ions in the UV/vis range, a Sunlite EX OPO tunable laser system (Continuum) is available. It is pumped with a seeded PL 9010 resulting in a line width <0.1 cm^−1^. The pulse length is 10 ns. The wavelengths of both OPO systems are calibrated using a WS6-200 HighFinesse wavelength meter.

The central aim of the present paper is to demonstrate that one can use the wide spectral output of a supercontinuum source for fast screening of ions in a wide spectral range. Radiation is generated using the supercontinuum laser (NKT Photonics, SuperK Extreme EXB-6, seed laser 1065.7 nm, 77.945 MHz). This laser provides the brightness of a laser with the bandwidth of a lamp in a single mode fiber. The total output power is 600 mW in the vis range. The spectral output power is important for spectroscopy (430–530 nm: 1.1 mW nm/600–680 nm: 1.7 mW nm, >730 nm: 1.7 mW nm). Using the acusto-optic tunable filter (AOTF) SuperK Select VIS/NIR AOTF we can vary the output between 420 and 720 nm. Over this range, the FWHM varies from 1.8 nm to 8.5 nm. The output power of this laser is measured using a Thorlabs power meter PM100A with the head S120C.

### Computational details

#### DFT calculations

All calculations were performed using the B3LYP functional^47^ and the 6-311+G(2d,p) basis set as implemented in the G16 program package. Geometries of the reported ions were fully optimized and are listed together with other computational results in Table S6 in the ESI.† The geometries of the singlet excited states were optimized using the TD-DFT approach.^48^ The vibronic spectrum of the anthracene dication in the visible range was calculated using the Franck–Condon method.^49^ The energy of the 0–0 transition was set to the experimental value (567.5 nm), because the theoretical excitation energy is 39 nm blue-shifted (to 528.5 nm). The infrared spectra were calculated in the harmonic approximation and scaled by a factor of 0.982 and in the anharmonic approximation (see the ESI†).

## 3. Results and discussion

Our approach for studying the structures of ions is based on the photodissociation spectroscopy of helium tagged ions in an ion trap. This method provides IR/vis/UV spectra of mass-selected cations with minimal perturbation caused by complexation with a helium atom. We have developed this method to study reactive intermediates in chemical reactions.^50^ However, we have demonstrated that the method can be applied in variety of research fields including applications in astrochemistry.^51^ Here, we will present in detail a top-down approach to study astrochemically relevant ions.

The usual approach in searching for possible DIBs carriers starts with an educated guess of a possible carrier and continues with the measurement of a sufficiently high-resolution spectrum in the range of the expected DIBs. We have decided to take an alternative approach to narrow down possible DIBs carrier candidates faster than when using usual spectra collection. The approach comprises 5 steps. (1) Generation of a series of cations by electron ionization or other methods, such as electrospray ionization. (2) Fast low-resolution scanning of the photodissociation spectra using helium tagging in the saturation regime. This step separates cations with sufficient absorption cross sections in the range of DIBs from those that can be discarded due to their low cross sections. This step usually requires only a few minutes. (3) Measurements of low resolution spectra with smaller photon fluence in order to identify possible bands. (4) Measurements of high resolution spectra. (5) Determination of photofragmentation cross sections. In the following, we will demonstrate the individual steps using cations generated from several polycyclic aromatic hydrocarbons.

### Generation of a series of cations by electron ionization

**Fig. 1:**
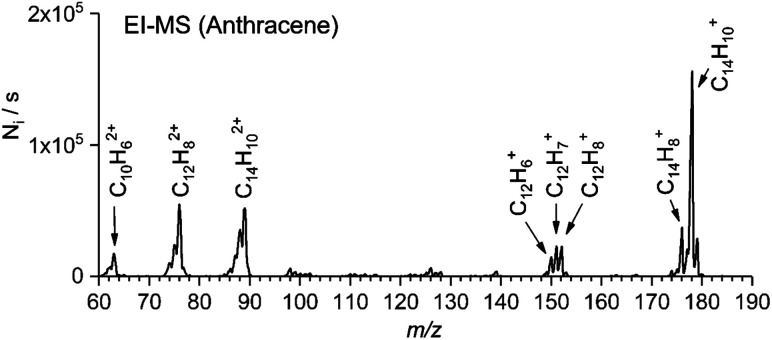
EI-MS spectrum of anthracene. The arrows denote which cations were selected for further screening.

Electron ionization (EI) of organic molecules provides a series of singly and multiply charged ions formed by ionization and subsequent fragmentation processes. As a typical example, we show a mass spectrum of anthracene obtained with an electron ionization source of TSQ using a solid probe (Fig. 1). From this EI-MS spectrum, we have selected eight abundant cations (five singly charged cations and three doubly charged cations) for further study. Similarly, we have generated parent ions and fragment ions from other aromatic hydrocarbon precursors (*e.g.* coronene, 1-pyrenecaboxylic acid, acridine, and others).

### Screening visPD spectra

**Fig. 2:**
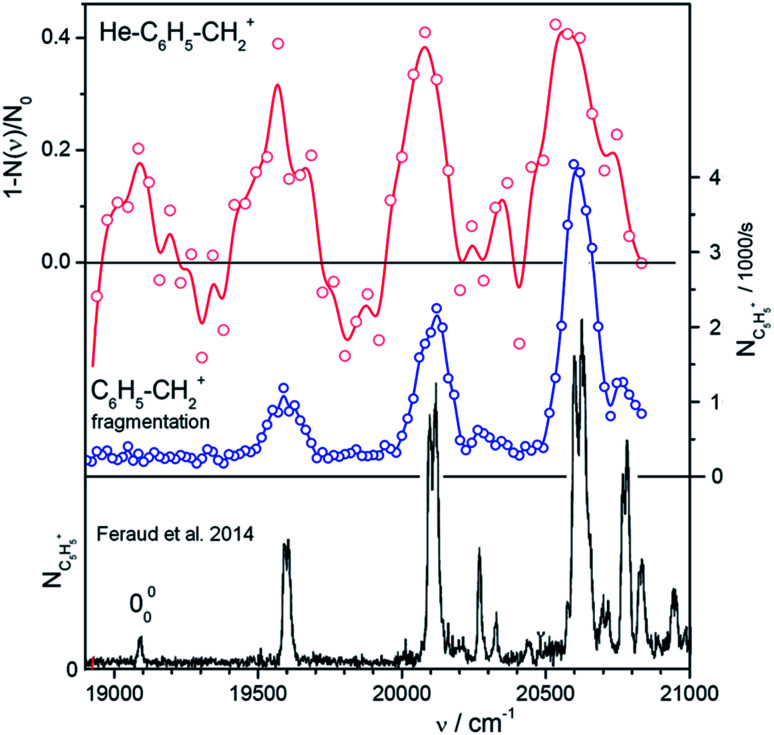
Vibronic spectra of benzylium ions in the range of the S_0_ and S_1_ transition. The ion C_6_H_5_–CH_2_^+^ has been well characterized using Ar tagging in a supersonic expansion^26^ and photofragmentation in a cold Paultrap.^30^ The spectrum at the bottom (black) has been taken from Fig. 1 of ref. 30. It shows *m*/*z* = 65 fragments scanned with an OPO with 8 cm^−1^ resolution. The upper two panels illustrate the efficiency of our method to get overview spectra using a SuperK laser. The upper trace (red) has been recorded using He-tagging. Due to inefficient tagging (only 200 complexes from 30 000 primaries per filling) the statistics are rather bad despite of an accumulation time of 20 min and an attenuation up to 40%. The data in the central panel (blue) have been recorded by photodissociating bare C_6_H_5_–CH_2_^+^ and detecting *m*/*z* = 65 fragments (12 iterations in 40 min). Note the narrow line width of only 3 nm in the middle spectrum. This indicates that a 2-photon fragmentation plays a role.

**Fig. 3:**
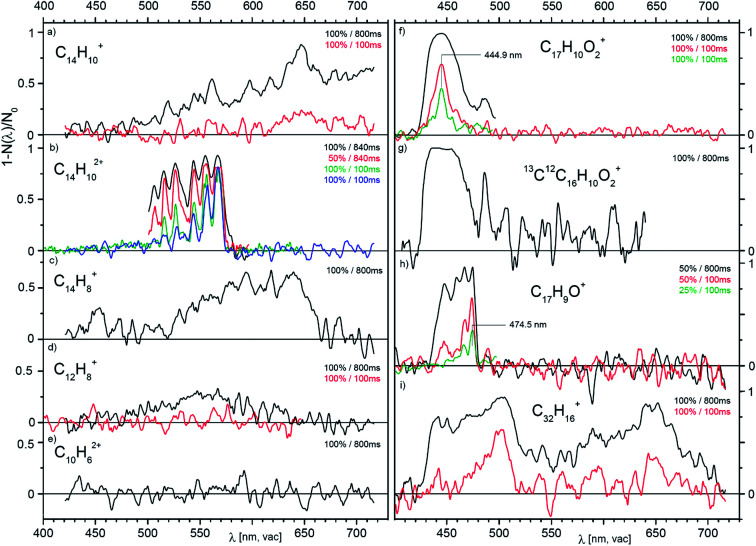
Overview photodissociation spectra of He-complexes of the indicated cations and dications generated by EI from anthracene (a–e), from 1-pyrenecaboxylic acid (f–h) and from dibenzo[7]helicene (i). The pairs of numbers in the upper right corners of the panels indicate the power level of the SuperK laser used (in %, see text) and the irradiation time (in ms). The data in (b) and (f) measured with the same levels of irradiation show that the trap conditions were slightly changing with time resulting in a change of the effective fluence (see discussion in the ESI†).

For rapid screening of where and how efficiently the mass-selected ions absorb in the visible range, we use a high-brilliance SuperK laser, as mentioned previously. Fig. 2, 3, and S3–S5 (see the ESI†) show helium tagging photodissociation spectra in the visible range for selected cations and dications generated by electron ionization from anthracene (Fig. 1) and other aromatic hydrocarbon molecules. Most of the studied cations and dications easily attached a helium atom in a simple timing sequence with one or two helium pulses (see Experimental details).

To illustrate the power of the method, we have investigated the region of the band head of benzylium ions (S_1_) (Fig. 2). This ion has been well characterized previously using Ar tagging in a supersonic expansion^26^ and photofragmentation in a cold Paul trap.^30^ We have generated the benzylium ions by electrospray ionization from benzylamine. Among the ions presented here, benzylium ions were exceptional due to their sluggish interaction with helium atoms. Nevertheless, a few generated helium complexes (only 200 per cycle from 30 000 primary ions) were sufficient to record an overview spectrum (Fig. 2a). We have also recorded a classical photodissociation spectrum by monitoring the *m*/*z* 65 fragments (Fig. 2b). While this inefficient helium tagging experiment represents a typical worst-case scenario for our technique, it still allows us to see the vibrational progression, which is in agreement with previously published results (Fig. 2c). This, and a total measuring time of just 1 hour for both spectra, provides confidence in our experimental strategy.

Fig. 3 presents a collection of data for ions from various precursors. Typically, an overview spectrum was recorded in 1 nm steps from 420 to 720 nm. At each point, we determine the number of trapped helium complexes with and without irradiation (2 cycles of 1 second each), which results in a measuring time of 10 min for one full scan. The first scan with the maximum laser fluence shows whether the selected ions have any absorption lines with a cross section above 10^−18^ cm^2^ (a typical result for a non-absorbing ion is shown in Fig. 3e). The interesting regions we further scanned in several iterations with 0.5 nm steps. This approach allowed us to collect all the reported screening spectra in six measuring days (see Table S1 in the ESI†).

The spectra shown in Fig. 3 point to several promising candidates for a detailed study. For high resolution studies, we have selected parent cations and dications generated from anthracene (Fig. 3a and b). Below, we will report a high resolution visPD spectrum of C_14_H_10_^2+^, a near IR absorption spectrum of C_14_H_10_^+^, and IRPD spectra of both C_14_H_10_^+^ and C_14_H_10_^2+^. Another promising candidate is C_17_H_9_O^+^ (Fig. 3h), the spectra for which features a resolved vibrational progression with a band head at 474.5 nm. This ion could be of interest as a DIBs carrier, but further study is necessary.

An interesting observation stems from panels f and g in Fig. 3. These panels compare the spectra of ^12^C_17_H_10_O_2_^+^ and ^13^C^12^C_16_H_10_O_2_^+^ (natural abundant isotope). Seemingly, replacing one ^12^C by ^13^C results in a larger photoabsorption cross section.

The last example (panel i) shows spectra at two irradiation times for the largest precursor studied here.^52^ In addition to the fragment ion C_32_H_16_^+^ shown, the parent ion (C_38_H_22_^+^), other fragments (*e.g.* C_38_H_18_^+^, C_32_H_16_^+^, C_31_H_15_^+^) and dications (C_32_H_16_^2+^, and C_38_H_18_^2+^) also show broad absorptions in the visible range (Fig. S4 in the ESI†). We have also screened ions generated from the nitrogen-containing aromatic compound acridine and ions generated from coronene (both in Fig. S5 in the ESI†). The spectra of the coronene dication features a narrow band at 559.7(2) nm and a broad absorption at 633(2) nm. The acridine dication absorbs in the vis range with a cross section of 5 × 10^−17^ cm^2^ and the vibrational progression has a band origin at 593 nm. For singly charged ions generated from acridine, the estimated absorption cross section is below 10^−18^ cm^2^.

### Low-resolution spectra of promising candidates

**Fig. 4:**
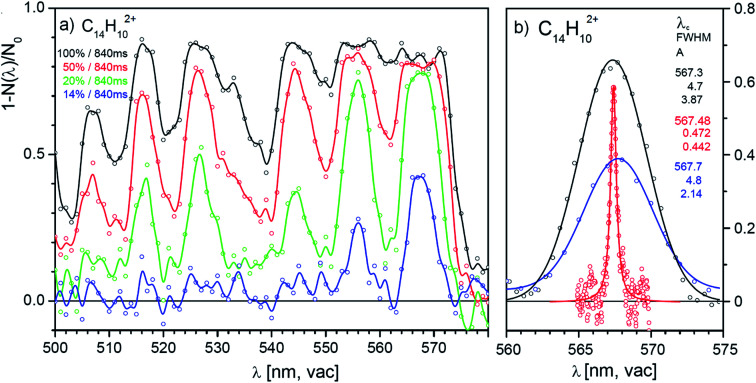
(a) Helium tagging visPD spectrum of C_14_H_10_^2+^ generated by electron ionization from anthracene. The spectra were recorded with different laser power levels (black: 100%; red: 50%; green: 20%; blue: 14%). (b) Comparison of line shapes measured under different conditions and with different laser sources. Blue: SuperK laser focused with lens; black: SuperK laser beam directly from the laser output; red: Sunlite laser. The numbers correspond to the indicated characteristics of the Gaussian (black and blue) and Lorentzian fits.

One of the promising candidates is the anthracene dication, we will therefore analyse the results for this dication in detail. Fig. 4a shows four selected scans of the absorption spectrum of the anthracene dication in the range from 500 nm to 600 nm measured with four different laser powers (1 nm step, the irradiation time was 840 ms and the diameter of the laser beam in the trap was ∼1 mm). The results show that with the full laser power (black line), helium complexes of anthracene dications are completely depleted over wide ranges. The fact that we do not reach 100% attenuation must be due to nonabsorbing ions present in the trap (either isomers with the same *m*/*z* ratio or ions with a different *m*/*z* ratio present due to imperfect mass resolution during mass-selection). Due to the oversaturation and nonlinear optical effects, the absorption lines become broad and the spectrum is not resolved. With decreasing laser power or shorter irradiation times, the absorption lines become better resolved and the positions of the absorption lines can be determined.

We have tested the reproducibility of the determination of the transition frequency of the detected bands in spectra measured with different laser intensities. Fig. 4b shows that the central position of the bands can be determined with ± 1 nm precision. The band is 0.2 nm red-shifted with respect to the band position determined in a much more demanding high-resolution experiment (see below). Table 1 lists the determined band positions and compares them with the results of our high-resolution experiment discussed below.

**Table 1:** Positions of the peaks in the vibronic spectrum of the anthracene dication determined from the measurements with SuperK and Sunlite lasers

SuperK		Sunlite	
*λ*_c_/nm	FWHM/nm	*λ*_c_/nm	FWHM/nm
506.9	4.9		
516.0	5.1	516.01	0.98
526.5	5.5	526.22	0.63
544.6	6.5	544.26	0.77
555.9	4.4	555.63	1.13
567.7	5.2	567.48	0.47

Hence, we can conclude that screening of the spectra with a low-resolution laser source provides two important results. First, the results identify ions that absorb in the studied range and importantly the results clearly show which ions are irrelevant for detailed studies. Second, measurements with different laser intensities provide a detailed overview of the absorption spectra with band positions with ± 1 nm precision.

### High-resolution spectra of promising candidates

We have outlined the procedure of screening for ions absorbing in the visible range and having absorption cross sections sufficiently large so that they could be considered as possible candidates for DIBs carriers. Next, we will show an in depth study of the anthracene dication.

**Fig. 5:**
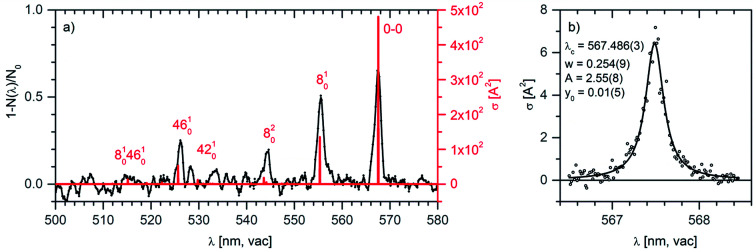
(a) Helium tagging visPD spectrum of C_14_H_10_^2+^ generated by electron ionization from anthracene measured with a Sunlite laser (see Experimental details). The red spectrum was calculated using the Franck–Condon harmonic approximation, and the 0–0 band position was set to the experimental value. (b) Details of the 0–0 transition band in the helium tagging visPD spectrum of C_14_H_10_^2+^. The parameters are characteristics of the Lorentzian fit. The Sunlite laser fluence was 0.7 mJ cm^−2^. For details of the absolute cross section determination see the ESI.†

Fig. 5a shows the vibronic spectrum of the anthracene dication measured with the high-resolution Sunlite OPO. The appearance of the spectrum with the largest intensity of the origin band suggests that the excited state has a similar geometry to the ground state. The next two lines correspond to the excitation to the first and second vibrationally excited state along a special normal mode coordinate (see the comparison with theory). Hence, it allows us to determine spectroscopic constants *ω*_e_ = 376.8 cm^−1^ and *ω*_e_*x*_e_ = 0.9 cm^−1^.

We have further analysed the origin band. After repetitive detailed screening of the band we concluded that the band can be fitted with a Lorentzian function with the line position at 567.486(3) nm and a width of 7.9(3) cm^−1^ (corresponding to 0.254 nm). The intensities at the tails of the bands can be considered as noise. Assuming that the broadening of the line is exclusively due to the lifetime broadening, one gets a lifetime of 0.67 ps. Rotation may contribute with 2 cm^−1^, which would not change much, and it would only make the lifetime slightly shorter.

The previous calculations as well as our theoretical analysis (see below) suggest that the anthracene dication should have another, even stronger transition in the UV range (around 270 nm). We have searched for this band, but we have detected only a broad peak at around 285 nm with a very low cross section (see Fig. S6 in the ESI†). This can be regarded as an important result for considering this dication of being of astrochemical relevance. The fact that it almost does not absorb in the UV range could have an impact on its lifetime in the interstellar medium.

#### Comparison with theory

We complement the experiments with DFT calculations. The vis-PD experiments showed that at a given transition with a sufficient laser fluence almost all helium complexes can be depleted. This indicates that the majority of the thermalised dications correspond to one isomer and one spin-state only. Hence, we have optimized the geometries of the anthracene dication in the singlet and the triplet state. The singlet state is 0.91 eV lower in energy than the triplet state, therefore, we have further considered the exclusive formation of the singlet state dications.

TD-DFT single-point calculations predict three electronic excitations with non-zero intensity. The lowest lying energy transition is predicted to the S_2_ state with a vertical excitation energy of 2.4558 eV (504.87 nm) and an oscillator strength *f* = 0.1999. The second active transition is predicted at 3.4283 eV (361.65 nm) with *f* = 0.0734 and corresponds to the transition to the S_3_ state. The most intense transition is predicted in the UV range at 4.6307 eV (267.74 nm) with *f* = 1.4569 and corresponds to the transition to the 9^th^ singlet excited state (all singlet vertical excitations are listed in Table S5 in the ESI†).

We have further analysed in detail the transition to the second excited state that corresponds to the progression that we have recorded in the visible spectral range. We have optimised the geometry of this state and calculated the vibronic spectrum using the Franck–Condon approximation. After optimisation of the geometry and inclusion of zero-point vibrational energies, the 0–0 transition shifted to 528.5 nm, which is still considerably blue-shifted with respect to the experimental data. For further evaluation, we have set the 0–0 transition to the experimental value of 567.5 nm. With this shift (without any additional scaling), the theoretical vibrational progression fits our experimental data very well (Fig. 5, the red stick spectrum). The transitions to vibrationally excited states of the S_2_ state are associated with the skeletal vibrations of the aromatic system. The mode 8, for which we determined experimental spectroscopic constants, has a harmonic frequency of 388 cm^−1^.

**Fig. 6:**
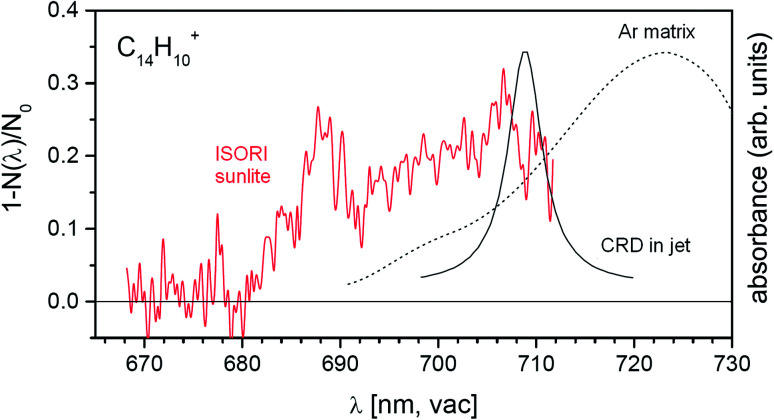
Comparison of the helium tagging vis-PD spectrum for anthracene monocations with previously obtained spectroscopic results for the same cations.^53,54^

The anthracene monocation has been previously studied using other spectroscopic methods. To demonstrate the sensitivity of our approach, we also include results for this ion, although the absorption intensity is weak. The previous experiments combining a supersonic jet with cavity ring-down spectroscopy unraveled the *D*_2_ ← *D*_0_ transition at 708.76 ± 0.13 nm.^53^ The same transition was also observed in argon matrix experiments, but the transition was red-shifted by 13.65 nm.^54^ The band position determined for this transition coincided with the position of a weak feature in the DIBs spectrum, but the measured width was too broad to comply with the astronomical observation. Using our approach, we clearly observe a band at about 689 nm (Fig. 6). Hence, using our highly sensitive approach we detect a weak spectral feature that remained hidden in the previous experiments.

### Determination of photoabsorption cross sections

**Fig. 7:**
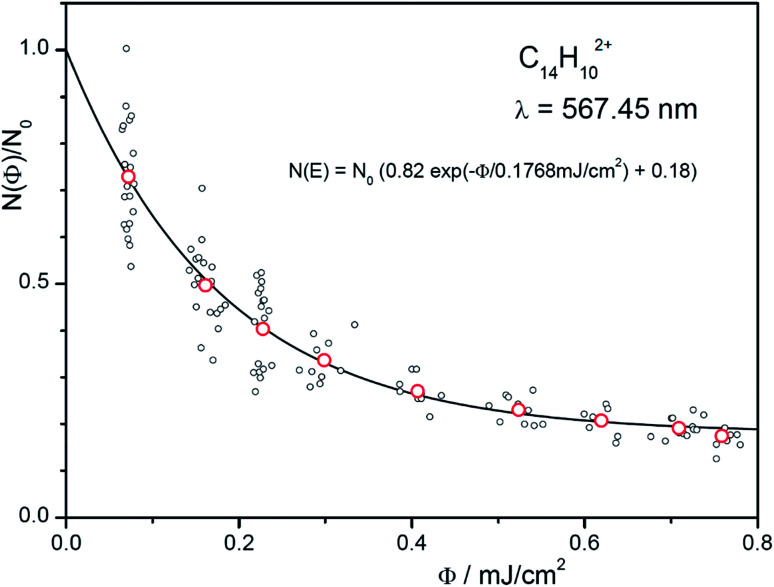
Number of complexes as a function of the fluence. The red points are the averages of the 10 iterations at each setting. The exponential fit function also includes non-absorbing ions (see eqn (S3) in the ESI†).

We will demonstrate how to determine absolute photoabsorption cross sections in helium tagging spectroscopy for the origin band at 567.48 nm in the anthracene dication vibronic spectrum. The cross section can be determined from the measurements of attenuation intensity as a function of laser fluence (see discussion and details in the ESI†).^46^ The fluence has been varied using neutral density filters. For each filter set, 10 measurements have been made (grey points in Fig. 7) and averaged (red points in Fig. 7). The data are fitted using eqn (S3)† resulting in *β* = 0.18 and *Φ*_0_ = 0.177 mJ cm^−2^. The parameter *β* accounts for ions that do not absorb light at the selected wavelength (most likely isomers or ions present due to imperfect mass-selection of the primary ions). Converting mJ into the number of photons (see ESI†) at the given wavelength leads to*σ* = 1.98 × 10^−15^ cm^2^.

The fluence *Φ* at the location of the ion trap is determined by measuring the energy per pulse at a reference point (here a beam splitter) and correcting it with a determined factor, corr (corr = 0.0103). This factor and the diameter, *d* (*d* = 1.8 mm), of the OPO beam (50% of the energy) are calibrated by deflecting it in front of the entrance of the instrument as shown in Fig. S2 (ESI†). In addition, one has to account for the number of pulses, *N*_pulses_ (*N*_pulses_ = 2).

The determined absorption cross section at 567.48 nm (19.8 A^2^) is one order of magnitude different to the theoretical cross section predicted for the 0–0 transition (481 A^2^).

### IRPD spectra

**Fig. 8:**
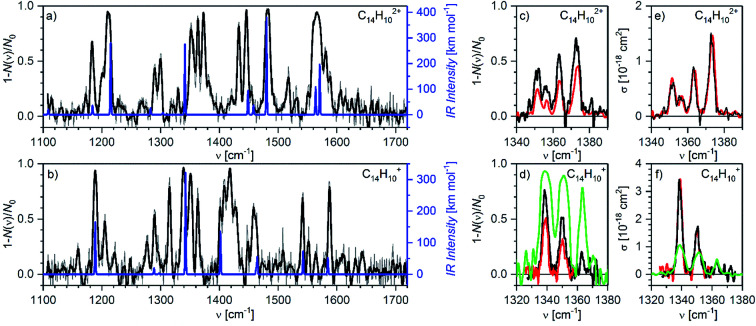
Helium tagging infrared photodissociation spectra of (a) C_14_H_10_^2+^ and (b) C_14_H_10_^+^ generated by electron ionization from anthracene. The black spectra are 10-point Savitzky–Golay smoothed original data (grey). The blue lines correspond to the B3LYP/6-311+G(2d,p) calculated harmonic vibrational spectra for the corresponding optimized ions. (c and d) Details of the bands in the range 1330–1380 cm^−1^ measured with different laser fluencies (green: 0.5 mJ with diameter *d* = 2.5 mm, black: 0.27 mJ with *d* = 2.8 mm, red: 0.13 mJ with *d* = 2.9 mm). (e and f) The same results as (c) and (d) but attenuation has been converted into cross section according to eqn (S6) (ESI†). It is obvious that this does not work anymore in the oversaturated regime.

Finally, we have also measured helium tagging infrared photodissociation spectra in the range from 1100 cm^−1^ to 1800 cm^−1^; thus, in the range of carbon–carbon stretching vibrations and C–C–H deformation vibrations (Fig. 8). We have collected the spectra using an OPO beam intensity of 1 mJ. The beam had 50% of the power in a diameter of 2.5 mm. Also in this case, the fluence was so high that we were operating in the saturation regime. We reached almost 100% attenuation of helium complexes of the anthracene dication as well as of the monocation at almost all fundamental bands (see the comparison with DFT calculated harmonic IR spectra in Fig. 8).

The large laser intensity enhanced fragmentation of helium complexes occurs at wavenumbers that result from non-linear effects and are not predicted at the harmonic approximation level (see the comparison with the theoretical IR spectra in Fig. 8). We have calculated anharmonic IR spectra, however, they did not provide a better agreement with the experimental results (Fig. S8 in the ESI†). The positions and widths of the peaks from both spectra are listed in Tables S3 and S4 (ESI†).

Fig. 8c and d show details of the bands in the range 1330–1380 cm^−1^. We have collected the spectra with several laser intensities (see the figure caption) and converted the attenuation to absolute cross section using eqn (S6).†Fig. 8d shows that measurements with a very high laser fluence lead to the saturation of the absorption bands and consequently to their large broadening (the green trace). Such data cannot be reliably used for cross section determination (Fig. 8f). At attenuations below 50% and with knowing the fraction of non-absorbing ions, cross sections can be calculated using eqn (S6)† with good reproducibility (black and red spectra in Fig. 7c–f).

## 4. Conclusions

The instrumental development in our laboratory and in parallel in other laboratories has pushed the limits of cryogenic ion traps to temperatures below 3 K and to buffer gas densities above 10^16^ cm^−3^. This resulted in the routine *in situ* synthesis of He-complexes from stored mass-selected ions and opened up photo-predissociation spectroscopy for reactive ions with applications in astrochemistry or bonding analysis. In astrochemical applications, the important experimental outcome is to identify ions with special optical properties, which usually requires scanning many members of a specific class of ions. Here, we demonstrate that the combination of a supercontinuum laser with our versatile instrument can be used to record many overview spectra localizing absorption lines with cross sections above 10^−18^ cm^2^ within a few minutes. The fact that, for He tagged ions, the photofragmentation cross section is equal to the photoabsorption cross section is an important basis for determining accurate absolute values.

As an outlook, we state that by further improving the stability of all instrumental components including the ion trap, the sensitivity can be increased by at least one order of magnitude, *i.e.* absorption lines with cross sections of 10^−19^ cm^2^ will be localized within a few minutes in the spectral range from 380 nm to 2.3 μm. The application can be rather broad, because the ions can be generated with different ionization techniques (electron ionization, electrospray ionization, laser ablation, and combustion sources) and can be further modified in the trap with suitable targets (*e.g.* H-atoms). Ion trapping also allows us to synthesize ions under inter- or circumstellar conditions. This makes the approach of helium tagging in a trap an ideal tool to search for astrochemically relevant ions.

In the present paper we screened spectra of dozens of ions and concentrated on the anthracene dication. It has a strong absorption in the visible range with well-resolved vibrational progression. In speculations about DIBs, we mention that some of the detected DIBs are close to the lines that we measured in the anthracene dication vis spectrum. Namely, DIBs no. 98 (5669.33 Å), no. 87 (5556.44 Å), no. 63 (5433.50 Å), no. 49 (5262.48 Å), and no. 40 (5170.49 Å) are rather close to the lines detected here (*cf.*Fig. 5 and see Table S2 in the ESI†). The fact that the second ionization energies of these larger aromatic hydrocarbons are well below the ionization energy of a hydrogen atom (13.6 eV) together with the observation that their absorption in the UV range might be negligible makes these types of dication good potential candidates for being DIBs carriers.

## Conflicts of interest

There are no conflicts to declare.

## Supplementary Material

FD-217-C8FD00196K-s001
