# Supplementary material for: Electronic spectra of ions of astrochemical interest: from fast overview spectra to high resolution
Source: Faraday Discuss. 2018 Dec 12;217:98–113. doi: 10.1039/c8fd00196k (PMC8639220; doi:10.1039/c8fd00196k)
Supplement: FD-217-C8FD00196K-s001 [file FD-217-C8FD00196K-s001.pdf]

Electronic Supplementary Material (ESI) for Faraday Discussions.
This journal is © The Royal Society of Chemistry 2018

ELECTRONIC SUPPORTING INFORMATION

for

Electronic spectra of ions of astrochemical interest: From fast overview spectra to high resolution

Jana Roithová,*[a] Juraj Jašík,[b] Jesus J. Del Pozo[a] and Dieter Gerlich[c]

a. *Institute for Molecules and Materials, Radboud University, Heyendaalseweg 135, 6525 AJ Nijmegen, Netherlands.*
b. *Department of Organic Chemistry, Faculty of Science, Charles University, Hlavova 2030/8, 128 43 Prague 2, Czech Republic.*
c. *Department of Physics, University of Technology, 09107 Chemnitz, Germany.*

Content

Experimental Setup, Figure S1 S2

Photodissociation of He-tagged ions in a w4PT, Figure S2 S2

Experimental Results

Additional data for "Screening visPD spectra" S6

(Figures S3 – S5)

Table S1 S9

UV-PD spectrum of anthracene dication (Figure S6) S12

Table S2 – S4 S12

Figure S7 S15

Theoretical Results

Results of TD-DFT calculations (Table S5) S16

Anharmonic IR spectra (Figure S8) S17

Geometries and energetics of calculated ions (Table S6) S18

Experimental Setup

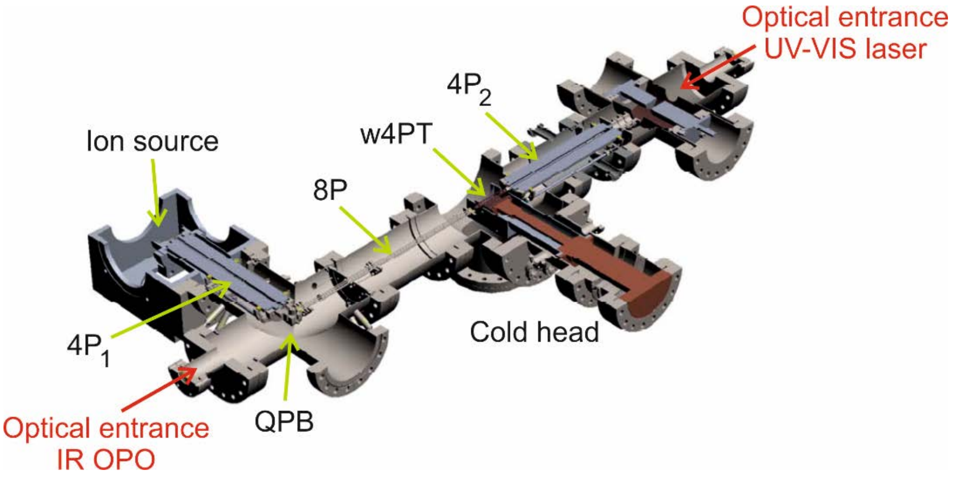

Figure S1. ISORI instrument. For details see: *Int. J. Mass Spectrom.* 2013, **354**, 204.

Photodissociation of He-tagged ions in a w4PT

Definition of σ

The frequency dependent photoabsorption cross section, σ, is usually defined via attenuation of a light beam passing an absorption cell containing the absorbing molecules at a density *n (see* Figure S2c). If I_0 is the intensity at the entrance, the intensity at the exit, *I,* is given by the Beer–Lambert law,

$$I = I_0 \exp(-\sigma\, n\, l\,). \tag{S1}$$

The intensity decays exponentially as a function of the product of the absorption cross section σ, the density *n* and the cell length *l*. The sensitivity of direct absorption techniques is primarily limited by intensity fluctuations of the light source requiring a relative intensity change of at least 0.001. The attenuation of light passing a trapped ion cloud is below 5×10^{-10}, assuming a cross section of 1 Å^2 and a column density of $n\, l$ ~ 5×10^{6}cm^{-2}. As an alternative, indirect absorption techniques have been developed that are based on monitoring an effect induced by the absorption of light ("action spectroscopy"). Such indirect techniques are many orders of magnitude more sensitive than direct absorption, but most of them do not provide directly absolute absorption cross sections.

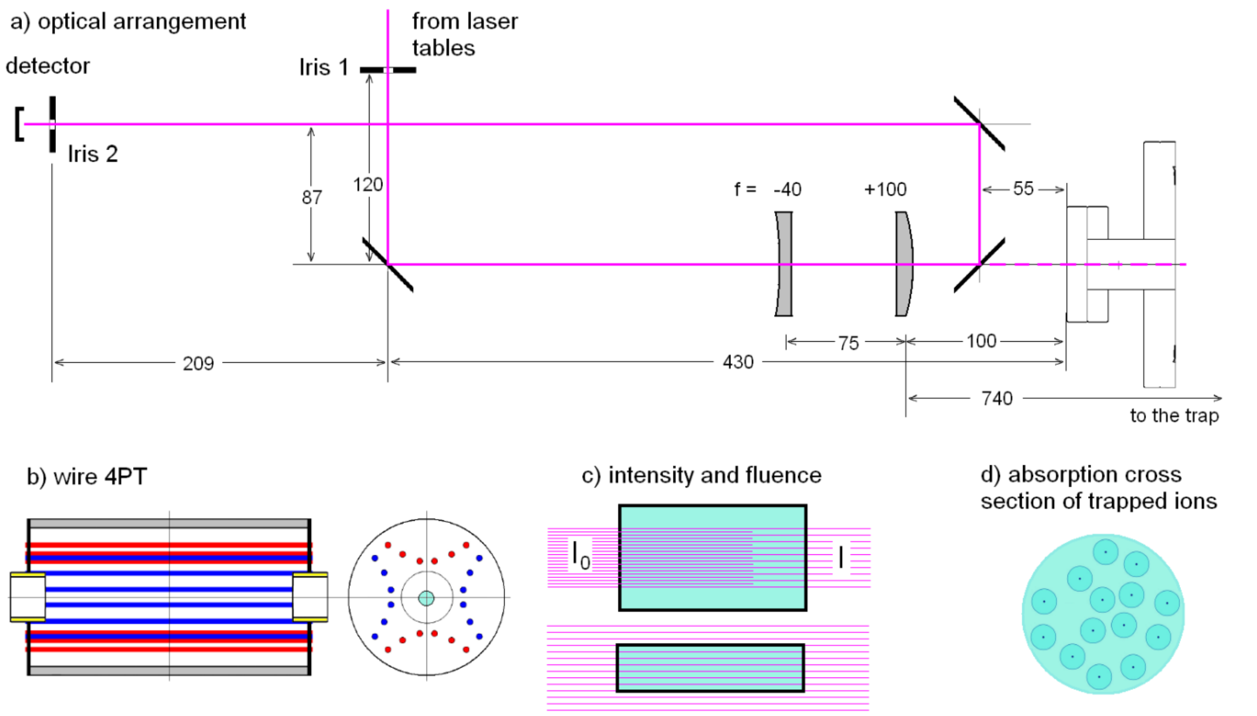

Figure S2. Determination of absolute photo absorption cross sections. Panel a) shows the optical arrangement to transfer the laser or OPO beam into the trap and to test the beam profile at this distance by reflecting it to the detector in the upper left corner. Panel b) shows the wire quadrupole trap that allows us to squeeze the ion cloud (blue point) into the light beam (magenta circle). The upper part of panel c) illustrates the definition of the photo absorption cross section via the Beer–Lambert law (Eq. S1) while the lower part depicts our trapping experiment, where a certain number of mass selected He-complexes is confined in a cylinder with a diameter below 1 mm and is exposed for a time interval (here up to 0.9 s) to the homogeneous flow of photons. Panel d) Simple geometrical illustration for deriving the probability to hit one of the trapped ions if one exposes them to a fluence of a certain number of photons/cm^2.

Our approach, based on He-tagging in a cryogenic w4PT, is an elegant method to obtain σ just from counting (typically every s) the number of the trapped He-tagged ions with and without laser. To optimize the geometry, the ions are confined in a cylinder with a small diameter (see figure S2b) and exposed to a homogeneous fluence of photons, Φ. Measuring alternatively the number of helium complexes with and without radiation, one gets

$$N(\Phi) = N_0 \exp(-\sigma\, \Phi). \qquad \text{(S2)}$$

This equation holds with the same cross section σ as introduced in Eq. 1, because the perturbation of the molecule by the attached He is negligible in most cases. Moreover, it is safe

to assume that, in the case of UV, vis or IR light, the absorption of a single photon always results in breaking the weak vdW bond.

The fluence Φ is the total number of photons per cm^2, the ion cloud has been exposed to. It is determined from the laser power or from the energy per OPO pulse, the irradiation time (or the number of light pulses) and from the geometry. In the present work we determine the mean fluence at the location of the trapped ions by deflecting the laser as illustrated in Fig. S2a and by measuring the effective diameter of the beam by closing iris 2 until the light detector shows 50% of the laser power (or energy). More accurate would be to permanently monitor the actual beam profile and its geometrical stability by reflecting a fixed fraction of the light beam. We also account for reflection and transmission loss by the mirrors and windows. In the present work, fluctuations of the light sources are taken into account by recording the energy per pulse (or power) behind the exit window of the instrument.

In practice, the number of complexes, $N(\Phi)$, remaining after exposing them to a laser fluence Φ, is described with the function

$$N(\Phi) = N_0\,((1-\beta)\,e^{-\frac{\Phi}{\Phi_0}} + \beta), \qquad \text{(S3)}$$

where the parameter β accounts for the fraction of trapped ions with the same mass which do not absorb light at the selected wavelength. In most cases these are isomers. The characteristic fluence, Φ_0, is determined at the central frequency of the peak, ν_c, by varying Φ (laser power or the irradiation time). A typical example is shown in Figure 6. Using the units cm^{-1} for ν and mJ/cm^2 for Φ_0 one gets σ in cm^2 from

$$\sigma = \nu / (conv\,\Phi_0) \qquad \text{(S4)}$$

with the conversion factor

$$conv = 0.001/1.602\times10^{-19} \times 8065.73 = 5.03423 \times 10^{19}\;. \qquad \text{(S5)}$$

For determining the cross section from a measured attenuation, one gets from Eqs. (S3) and (S4)

$$\sigma = -\frac{\nu}{conv\,\Phi}\ln\left[(\frac{N(\Phi)}{N_0} - \beta)/(1-\beta)\right] \qquad \text{(S6)}$$

As already mentioned above, $conv\times\Phi_0/\nu$ is the number of photons per cm^2, the ions have been exposed to. With the help of Figure S2d, a simple statistical consideration allows one to proof that σ is really the absorption cross section.

Accuracy and problems

In principle, our method to record absolute cross sections is not only fast (measurements like the one shown in Figure 6 can be done in less than 10 min) but it is also very precise, at least in principle. In an ideal linear quadrupole, the effective potential of the quadrupole trap can squeeze the ions into a cylinder with a diameter of 0.25 mm ($C_{14}H_{10}^{2+}$, 1 MHz, V_0 = 100 V). With the geometry shown in Fig. S2 the SuperK laser has been focused to a diameter of 0.35 mm resulting in a maximum spectral fluence of 10 mJ per cm^2 and per nm. With this fluence it is easy to fully saturate many transitions as shown in this paper.

In practice, several experimental difficulties hamper obtaining precise values of absolute cross sections. The measurements depend on the geometry of all relevant optical parts (especially the pointing stability of the light sources) which have to be therefore very stable. Also, we had problems with the precise localization of the ion cloud in some of our experiments. For example, small potential perturbations of the trap electrodes can push (or pull) the ion cloud away from the geometrical center line of the trap and reduce the effective fluence. The results in Fig. 2b and 2f show measurements with the same settings of the laser; however, within 2 hours, the effective fluence has been reduced. Readjustment of the relevant parameters can usually compensate the perturbations. In the case of surface problems ("patch effects") usually the electrodes have to be cleaned. The trapping conditions can also be improved for a few hours by turning off the cold head and warming up the electrodes to 50 K. It is important to mention that space charge effects play a role if one operates with ion densities of more than 10^6 ions/cm^3.

Under unfavorable conditions our measured absolute cross sections could differ by up to a factor 3. Usually, for a fixed arrangement of the laser etc. the results are reproducible with a statistical error of smaller than 5%. At the moment, this can be completely neglected in comparison with the systematic errors. In the present arrangement, the determination of the effective fluence is certainly the biggest problem, typically up to 50%. Errors from the calibrated laser power meter (< 5%) can be neglected. Most problematic is the exact position and the shape of the laser profile inside the trap and its overlap with the ions cloud. For reducing these uncertainties, we have increased the distance of the two lenses (from 75 to 77 mm), shifting the focus out of the trap and increasing the diameter of the slightly divergent light beam to 1.1 mm. This results in more stable conditions; however, it reduces the fluence by a factor of 10. Finally, the relative number of ions which do not absorb (β in Eq. 3), can introduce an error of up to 10 %. Overall, the results presented in this paper, have an uncertainty of up to a factor 2. Improving the experiment (cleaner or other trap electrodes, better optics, e.g. a beam profiler), our method can provide absolute cross sections with an accuracy better than 10%. This is also possible with the SuperK laser. The only additional uncertainty is the actual line width of the beam prepared with the AOTF filter.

Experimental Results

Additional data to “Screening visPD spectra”

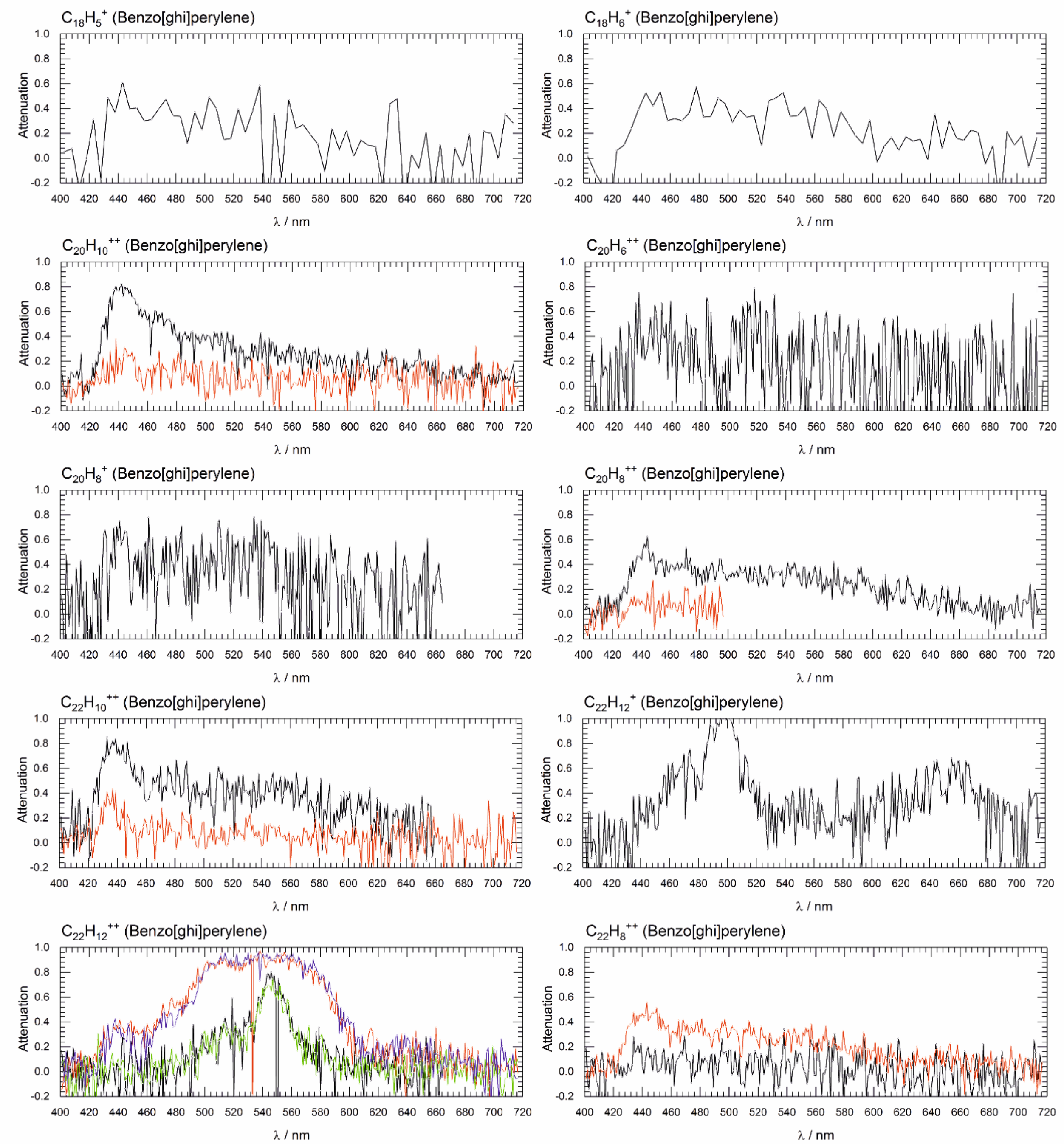

Figure S3. Overview photodissociation spectra of He-complexes of the indicated cations and dications generated by EI from benzo[ghi]perylene. Spectra recorded with different power levels of the SuperK laser are colour-coded.

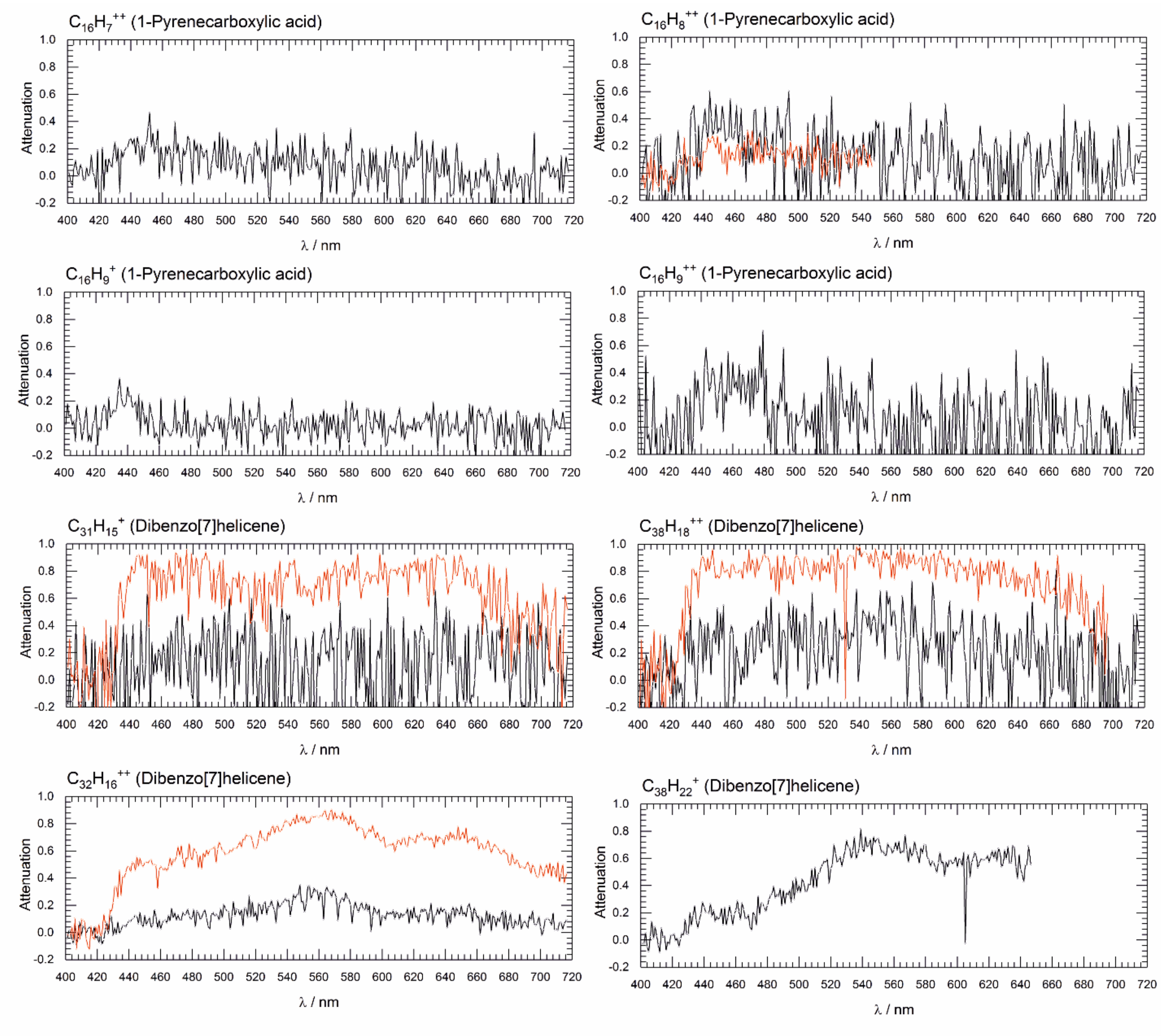

Figure S4. Overview photodissociation spectra of He-complexes of the indicated cations and dications generated by EI from 1-pyrenecarboxylic acid and dibenzo[7]helicene. Spectra recorded with different power levels of the SuperK laser are colour-coded.

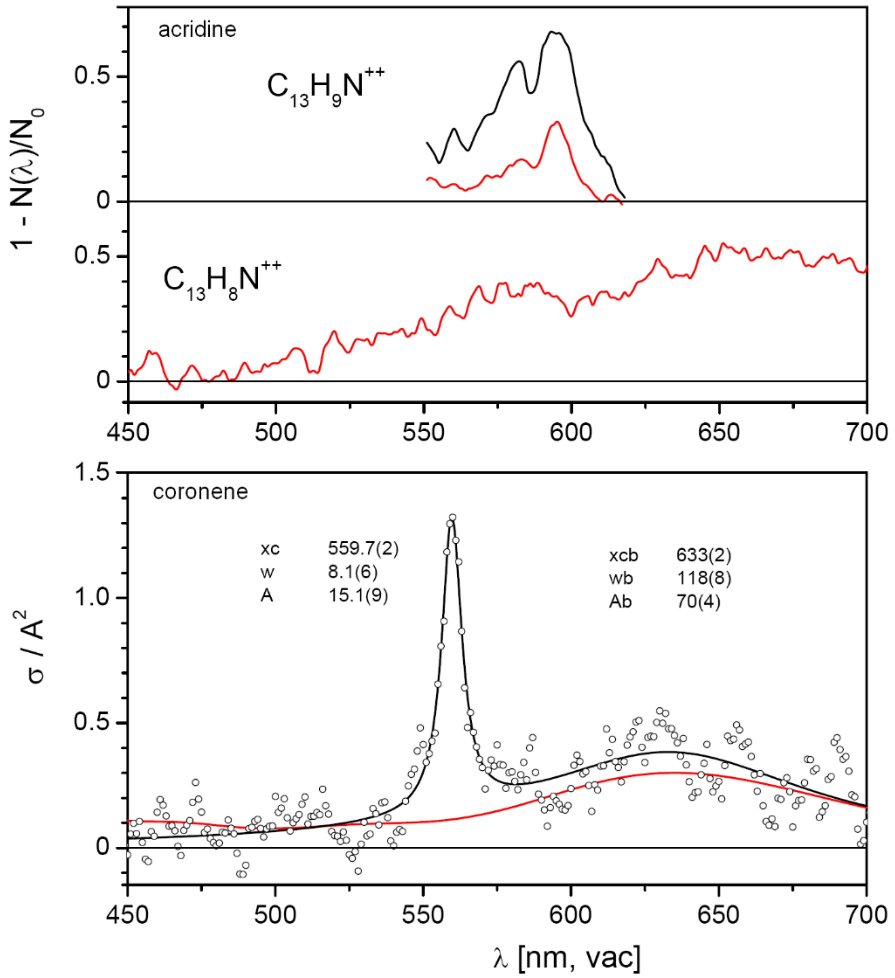

Figure S5. Helium tagging vis-PD spectra of $C_{13}H_9N^{2+}$ and $C_{13}H_8N^{2+}$ generated from acridine (upper panel) and $C_{24}H_{12}^{2+}$ generated from coronene.

Table S1. List of helium tagging vis-PD spectra measured for ions generated from different aromatic hydrocarbon precursors.

Nr	m/z	Ion	Date	File	PO / %	RF / %	Dt / ms	n / nm	Dn /nm	att / %	fig	Note
**1-Pyrenecarboxylic acid**												
1	247	$^{13}C^{12}C_{16}H_{10}O_2^+$	19.6.2018	vis11	100	20	800				2g	
2	246	$C_{17}H_{10}O_2^+$	19.6.2018	vis06	100	20	100	445.0	21	62	2f	P=45uW
3	246	$C_{17}H_{10}O_2^+$	19.6.2018	vis07	50	20	100					
4	246	$C_{17}H_{10}O_2^+$	19.6.2018	vis08	100	30	100	444.5	18	76		
5	246	$C_{17}H_{10}O_2^+$	19.6.2018	vis09	100	20	100	444.9	18	36	2f	
6	246	$C_{17}H_{10}O_2^+$	19.6.2018	vis10	100	20	800				2f	
7	229	$C_{17}H_9O^+$	20.6.2018	vis01	100	20	100					
8	229	$C_{17}H_9O^+$	20.6.2018	vis02	100	20	100					P 100 and 50%
9	229	$C_{17}H_9O^+$	19.6.2018	vis12	50	20	800				2h	
10	229	$C_{17}H_9O^+$	19.6.2018	vis13	25	20	100	466.3	4	28		
11	229	$C_{17}H_9O^+$	19.6.2018	vis13	25	20	100	474.5	3	54	2h	
12	229	$C_{17}H_9O^+$	19.6.2018	vis14	50	20	100	466.9	5	50		
13	229	$C_{17}H_9O^+$	19.6.2018	vis14	50	20	100	474.3	3	93		
14	229	$C_{17}H_9O^+$	19.6.2018	vis15	50	20	100				2h	
15	201	$C_{16}H_9^+$	19.6.2018	vis01	100	20	800	438.0	20	23		
16	101	$C_{16}H_9^{++}$	19.6.2018	vis05	100	20	800	462.0	40	35		
17	100	$C_{16}H_8^{++}$	19.6.2018	vis02	100	20	800					
18	100	$C_{16}H_8^{++}$	19.6.2018	vis03	100	20	800	470.0	b	20		
19	99.5	$C_{16}H_7^{++}$	19.6.2018	vis04	100	20	800	450.0	b	20		
**Anthracene**												
**Nr**	**m/z**	**Ion**	**Date**	**File**	**PO / %**	**RF / %**	**Dt / ms**	**n / nm**	**Dn / nm**	**att / %**	**fig**	**Note**
20	178	$C_{14}H_{10}^+$	13.6.2018	vis01	100	20	840					
21	178	$C_{14}H_{10}^+$	13.6.2018	vis02	100	20	840					
22	178	$C_{14}H_{10}^+$	14.6.2018	vis17	100	20	800				2a	
23	178	$C_{14}H_{10}^+$	14.6.2018	vis18	100	20	100				2a	
24	176	$C_{14}H_8^+$	14.6.2018	vis10	100	20	800				2c	580 - 640nm att 60%
25	152	$C_{12}H_8^+$	14.6.2018	vis08	100	20	100				2d	no line, att=0.8±13%
26	152	$C_{12}H_8^+$	14.6.2018	vis09	100	20	800	554.0	100	25	2d	
27	151	$C_{12}H_7^+$	14.6.2018	vis06	100	20	100					no line att=-2±6.7%
28	150	$C_{12}H_6^+$	14.6.2018	vis07	100	20	100					no line, att=-0.3±8%
29	101	$C_8H_5^+$	14.6.2018	vis14	100	20	800					no line, att=-5±31%

30	100	$C_{8}H_{4}^{+}$	14.6.2018	-	-	-	-					no He complexes
31	99	$C_{8}H_{3}^{+}$	14.6.2018	-	-	-	-					no He complexes
32	98	$C_{8}H_{2}^{+}$	14.6.2018	-	-	-	-					no He complexes
33	89	$C_{14}H_{10}^{++}$	13.6.2018	vis03	100	20	840				2b	
34	89	$C_{14}H_{10}^{++}$	14.6.2018	vis01	100	20	840				3	
35	89	$C_{14}H_{10}^{++}$	14.6.2018	vis02	50	20	840				2b,3	
36	89	$C_{14}H_{10}^{++}$	14.6.2018	vis03	20	20	840				3	
37	89	$C_{14}H_{10}^{++}$	14.6.2018	vis04	14	10	840				3	$\sigma = 5.5x10^{-16}$ cm^{2}
38	89	$C_{14}H_{10}^{++}$	14.6.2018	vis05	100	20	100				2b	
39	89	$C_{14}H_{10}^{++}$	14.6.2018	vis11	100	20	100				2b	
40	89	$C_{14}H_{10}^{++}$	14.6.2018	vis16	100	20	100					
41	76	$C_{12}H_{8}^{++}$	14.6.2018	vis13	100	20	800	550.0	200	9		
42	63	$C_{10}H_{6}^{++}$	14.6.2018	vis15	100	20	800				2e	no line, att=0.4±19%
**Benzo[ghi]perylene**												
**Nr**	**m/z**	**Ion**	**Date**	**File**	**PO / %**	**RF / %**	**Dt / ms**	**n / nm**	**Dn / nm**	**att / %**	**fig**	**Note**
43	276	$C_{22}H_{12}^{+}$	20.6.2018	vis03	100	20	800	465.0	33	50		
44	276	$C_{22}H_{12}^{+}$	20.6.2018	vis03	100	20	800	498.0	27	87		
45	276	$C_{22}H_{12}^{+}$	20.6.2018	vis03	100	20	800	550.0	97	23		
46	276	$C_{22}H_{12}^{+}$	20.6.2018	vis03	100	20	800	653.0	68	50		
47	248	$C_{20}H_{8}^{+}$	21.6.2018	vis12	100	20	800	440.3	18	64		
48	222	$C_{18}H_{6}^{+}$	21.6.2018	vis10	100	20	800					440 - 580nm att 40%
49	221	$C_{18}H_{5}^{+}$	21.6.2018	vis11	100	20	800					430 - 570 nm att 35%
50	138	$C_{22}H_{12}^{++}$	21.6.2018	vis01	100	20	800					
51	138	$C_{22}H_{12}^{++}$	21.6.2018	vis02	100	20	100	508.0	29	26		
52	138	$C_{22}H_{12}^{++}$	21.6.2018	vis02	100	20	100	548.0	35	62		
53	138	$C_{22}H_{12}^{++}$	20.6.2018	vis04	100	20	800					
54	138	$C_{22}H_{12}^{++}$	20.6.2018	vis05	100	20	100					
55	137	$C_{22}H_{10}^{++}$	21.6.2018	vis03	100	20	100	436.6	20	31		
56	137	$C_{22}H_{10}^{++}$	20.6.2018	vis06	100	20	800					
57	136	$C_{22}H_{8}^{++}$	21.6.2018	vis04	100	20	800					
58	136	$C_{22}H_{8}^{++}$	21.6.2018	vis05	100	20	100					att < 20%
59	125	$C_{20}H_{10}^{++}$	21.6.2018	vis06	100	20	800	442.0	24	80		
60	125	$C_{20}H_{10}^{++}$	21.6.2018	vis06	100	20	100	442.8	21	24		
61	124	$C_{20}H_{8}^{++}$	21.6.2018	vis07	100	20	800	442.3	24	50		
62	124	$C_{20}H_{8}^{++}$	21.6.2018	vis08	100	20	100					att < 20%
63	123	$C_{20}H_{6}^{++}$	21.6.2018	vis09	100	20	800	453.0	41	43		
**Dibenzo[7]helicene**												

Nr	m/z	Ion	Date	File	PO / %	RF / %	Dt / ms	n / nm	Dn / nm	att / %	fig	Note
64	479	$C_{38}H_{22}^{+}$	22.6.2018	vis01	100	20	800	450.0		20		
65	479	$C_{38}H_{22}^{+}$	22.6.2018	vis01	100	20	800	550.0		70		
66	401	$C_{32}H_{16}^{+}$	22.6.2018	vis03	100	20	800				2i	
67	401	$C_{32}H_{16}^{+}$	22.6.2018	vis04	100	20	100	497.0	32	54	2i	
68	401	$C_{32}H_{16}^{+}$	22.6.2018	vis04	100	20	100	651.0	40	27		
69	387	$C_{31}H_{15}^{+}$	22.6.2018	vis05	100	20	800					430 - 650 nm att 75%
70	387	$C_{31}H_{15}^{+}$	22.6.2018	vis06	100	20	100					
71	237	$C_{38}H_{18}^{++}$	22.6.2018	vis08	100	20	800					
72	237	$C_{38}H_{18}^{++}$	22.6.2018	vis09	100	20	100					broadband att 40%
73	200	$C_{32}H_{16}^{++}$	22.6.2018	vis07	100	20	800					
74	200	$C_{32}H_{16}^{++}$	22.6.2018	vis07	100	20	100	560.0	60	30		broadband att

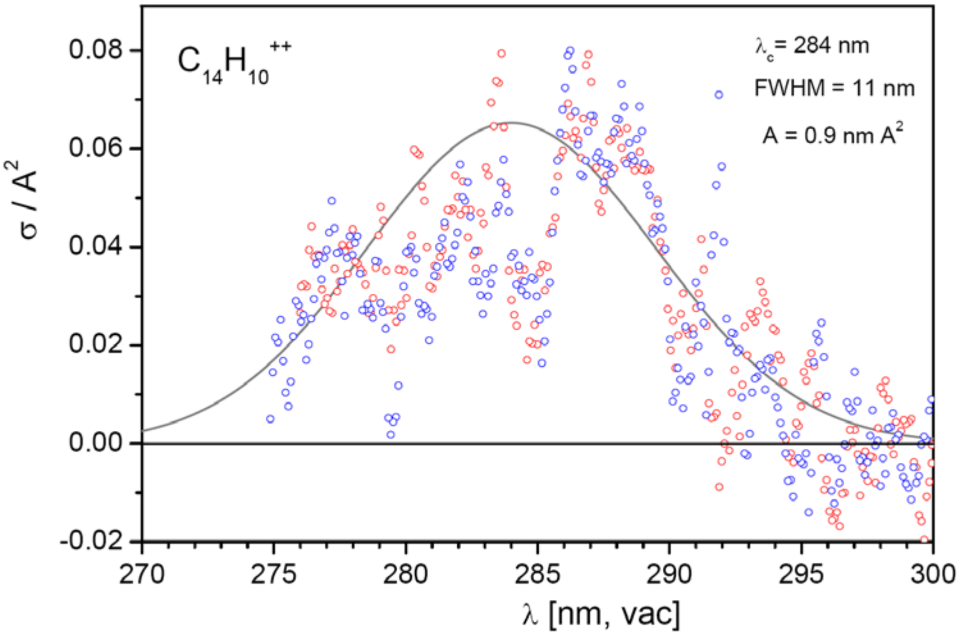

Figure S6. UV absorption of the anthracene dication. The spectrum features a very broad (~10 nm) absorption band around 285 nm. The cross section is below 0.1 Å^2.

Table S2. Comparison of the vibronic spectrum of anthracene dication with DIBs.

**DIB data**			**$C_{14}H_{10}^{2+}$**		**Difference/Å**
**DIB number**	**l_c / Å**	**FWHM /Å**	**exp/Å**	**fwhm exp/Å**	
40	5170.49	0.47	5160.11	9.8	10.38
49	5262.48	0.54	5262.20	6.3	0.28
63	5433.50	0.45	5442.56	7.7	-9.06
87	5556.44	1.28	5556.26	11.3	0.18
98	5669.33	1.47	5674.80	2.54	-5.47

Position of bands in helium tagging IRPD spectra of anthracene dication and monocation

Table S3. Peak positions in helium tagging IRPD spectrum of anthracene dication. The centers, heights and FWHM values were determined from fitting the measured IRPD spectra with Gaussian functions (see Figure S8).

Nr	Center	Height	Area	FWHM
1	1172.73	0.25	1.00	3.8
2	1183.63	0.70	4.81	6.4
3	1199.70	0.35	2.54	6.7
4	1211.11	1.02	11.12	10.3
5	1269.63	0.13	1.60	11.8
6	1290.07	0.47	2.94	5.8
7	1299.51	0.58	3.48	5.7
8	1320.25	0.09	0.88	8.8
9	1329.95	0.29	1.10	3.6
10	1351.59	0.85	5.89	6.5
11	1356.73	0.54	1.70	2.9
12	1363.44	0.95	5.00	4.9
13	1372.73	0.83	5.29	6.0
14	1381.35	0.27	5.44	18.7
15	1422.48	0.14	3.43	22.8
16	1433.25	0.82	4.44	5.1
17	1445.92	0.99	7.15	6.8
18	1454.81	0.29	1.06	3.4
19	1482.21	1.01	11.62	10.9
20	1516.55	0.33	2.86	8.0
21	1566.11	0.99	19.50	18.5
22	1581.45	0.48	2.50	4.9
23	1588.62	0.37	2.14	5.4

Table S4. Peak positions in helium tagging IRPD spectrum of anthracene monocation. The centers, heights and FWHM values were determined from fitting the IRPD spectrum with Lorentzian functions.

Nr	Center	Height	Area	FWHM
26	1159.25	0.25	1.33	4.9
27	1188.95	0.98	7.63	7.3
28	1205.04	0.46	3.59	7.4
29	1221.41	0.22	1.32	5.7
30	1276.63	0.28	2.36	7.8
31	1289.68	0.62	3.23	4.9
32	1295.69	0.37	0.86	2.2
33	1315.23	0.93	4.49	4.6
34	1339.23	1.00	9.07	8.5
35	1350.97	0.97	7.17	6.9
36	1363.40	0.77	3.77	4.6
37	1401.25	0.72	3.23	4.2
38	1407.86	0.77	4.14	5.0
39	1417.26	0.97	10.77	10.4
40	1428.47	0.58	4.78	7.8
41	1447.69	0.49	1.12	2.2
42	1459.38	0.61	6.34	9.7
43	1541.59	0.80	3.63	4.2
44	1551.59	0.39	1.46	3.6
45	1564.41	0.22	1.02	4.4
46	1587.54	0.88	4.56	4.9

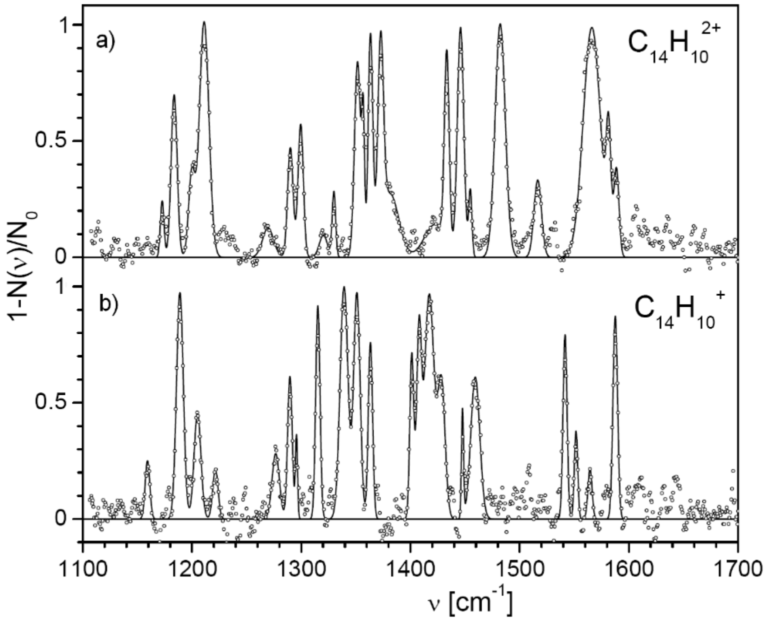

Figure S7. Helium tagging infrared photodissociation spectra of $C_{14}H_{10}^{2+}$ (upper panel) and $C_{14}H_{10}^{+}$ (lower panel) generated by electron ionization from anthracene. The experimental data (points) were fitted with Gaussian peaks. The characteristics of the fits are summarized in Tables S3 and S4.

Results of DFT calculations

Table S5. TD-DFT (B3LYP/6-311G+(2d,p)) results for singlet state excitations.

State (symmetry)		Excitation energy [eV]	Excitation energy [nm]	Oscilator strength[a]
S_1	Singlet-A'	1.4464	857.22	f=0.0000
S_2	Singlet-A'	2.4558	504.87	f=0.1999
S_3	Singlet-A'	3.4283	361.65	f=0.0734
S_4	Singlet-A'	3.6674	338.07	f=0.0000
S_5	Singlet-A"	3.7153	333.71	f=0.0000
S_6	Singlet-A"	3.7879	327.31	f=0.0000
S_7	Singlet-A'	4.0739	304.34	f=0.0000
S_8	Singlet-A"	4.1568	298.27	f=0.0000
S_9	Singlet-A'	4.6307	267.74	f=1.4569
S_{10}	Singlet-A"	4.6409	267.16	f=0.0004

[a] Definition of oscilator strength:

$$f_i = \frac{8\pi^2 \tilde{v}_i m_e c}{3he^2} D_i$$

where f_i is the (dimensionless) oscillator strength corresponding to the electronic excitation of interest, and D_i is the corresponding dipole strength in $esu^2\ cm^2$; ṽ is the corresponding excitation energy in wavenumbers. The remaining two constants are the charge on the electron: $e = 4.803204 \times 10^{-10}$ esu and the electron mass: $m_e = 9.10938 \times 10^{-31}$ kg.

Anharmonic theoretical IR spectra

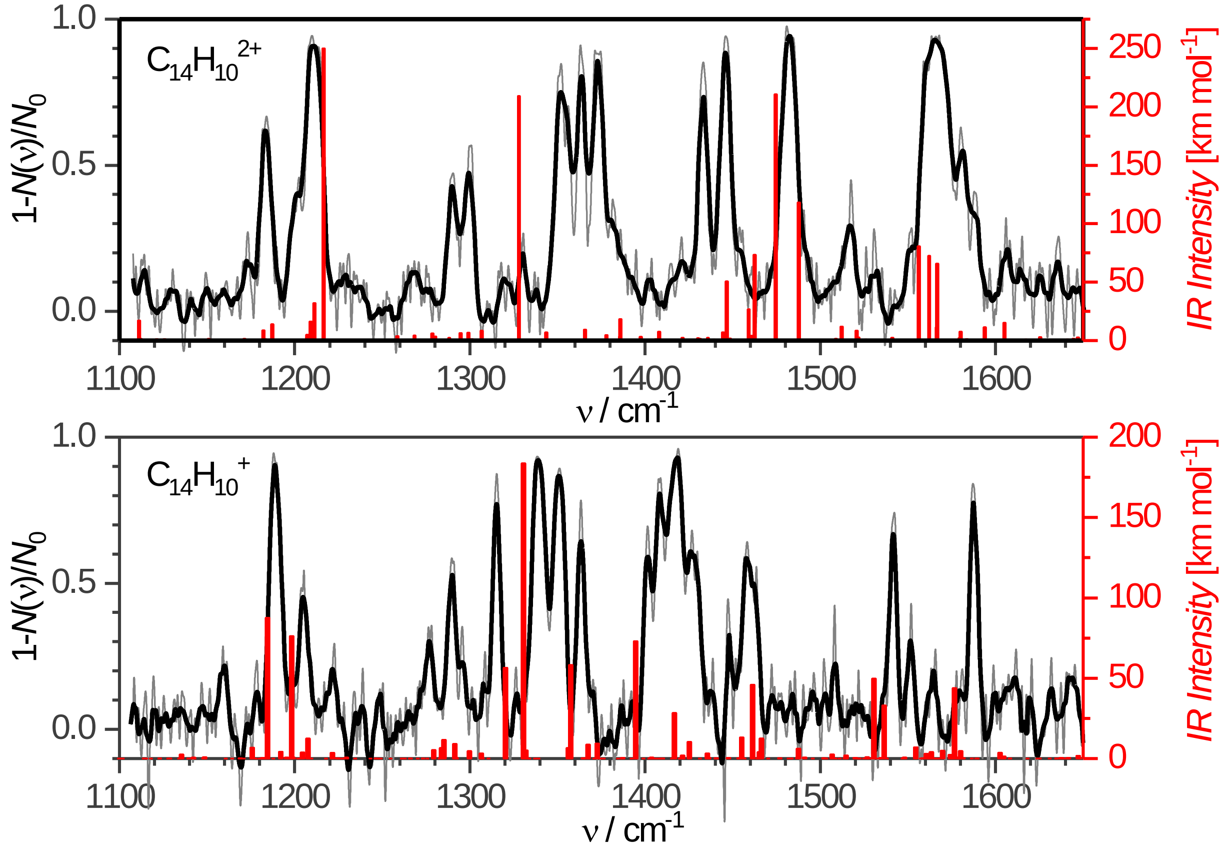

Figure S8. Helium tagging infrared photodissociation spectra of $C_{14}H_{10}^{2+}$ (upper panel) and $C_{14}H_{10}^{+}$ (lower panel) generated by electron ionization from anthracene. The black spectra are 5-point smoothed original data (grey). The red stick spectra correspond to the B3LYP/6-311+G(2d,p) calculated anharmonic vibrational spectra for the corresponding optimized ions.

Table S6. Optimized geometries and energetics of anthracene dication in the S_0, T_1, S_2 and S_3 states and anthracene monocation. Method B3LYP/6-311G+(2d,p), the S_2 state was optimized using TD DFT approach.

Ion, State, energetics, FC simulation	Optimized geometry

$C_{14}H_{10}^{2+}$: S_0

(E_{rel} = 0.00 eV)

Charge = 2 Multiplicity = 1

Low frequencies --- -0.0003 0.0003 0.0004 2.8318 3.7989 6.4231
Low frequencies --- 79.5717 117.3439 198.9757

Zero-point correction= 0.193544 (Hartree/Particle)
Thermal correction to Energy= 0.203308
Thermal correction to Enthalpy= 0.204252
Thermal correction to Gibbs Free Energy= 0.158367
Sum of electronic and zero-point Energies= -538.791583
Sum of electronic and thermal Energies= -538.781820
Sum of electronic and thermal Enthalpies= -538.780876
Sum of electronic and thermal Free Energies= -538.826761

Center Number	Atomic Number	Atomic Type	Coordinates (Angstroms) X	Y	Z
1	6	0	-0.013176	0.000000	0.026256
2	6	0	0.008215	0.000000	1.426808
3	6	0	1.236432	0.000000	2.122175
4	6	0	2.432709	0.000000	1.426038
5	6	0	2.434956	0.000000	0.014645
6	1	0	-0.922272	0.000000	1.984084
7	1	0	1.235547	0.000000	3.205681
8	1	0	3.375174	0.000000	1.960591
9	1	0	3.379183	0.000000	-0.519019
10	6	0	-1.227650	0.000000	-0.691497
11	6	0	-1.251652	0.000000	-2.102000
12	6	0	-0.010641	0.000000	-2.824171
13	6	0	1.203837	0.000000	-2.106419
14	6	0	1.227840	0.000000	-0.695917
15	1	0	-2.166419	0.000000	-0.145209
16	6	0	-2.458771	0.000000	-2.812562
17	1	0	2.142604	0.000000	-2.652709
18	6	0	-2.456527	0.000000	-4.223954
19	1	0	-3.402995	0.000000	-2.278895
20	1	0	-3.398990	0.000000	-4.758507
21	6	0	-1.260250	0.000000	-4.920093
22	6	0	-0.032032	0.000000	-4.224727
23	1	0	-1.259367	0.000000	-6.003598
24	1	0	0.898455	0.000000	-4.782000

$C_{14}H_{10}^{2+}$: T_1
Charge = 2 Multiplicity = 3

(E_{rel} = 0.91 eV)

Low frequencies --- -5.2739 -3.8162 -2.2506 -0.0004 -0.0002 0.0003
Low frequencies --- 81.6851 104.3193 203.7275

Zero-point correction= 0.190551 (Hartree/Particle)

Center Number	Atomic Number	Atomic Type	Coordinates (Angstroms) X	Y	Z
1	6	0	-0.004945	0.000000	0.007087
2	6	0	0.013125	0.000000	1.453152
3	6	0	1.201788	0.000000	2.138345
4	6	0	2.421149	0.000000	1.426645
5	6	0	2.443578	0.000000	0.013408
6	1	0	-0.930486	0.000000	1.986941
7	1	0	1.212238	0.000000	3.221335

Thermal correction to Energy= 0.200842
Thermal correction to Enthalpy= 0.201787
Thermal correction to Gibbs Free Energy= 0.153860
Sum of electronic and zero-point Energies= -538.758173
Sum of electronic and thermal Energies= -538.747882
Sum of electronic and thermal Enthalpies= -538.746938
Sum of electronic and thermal Free Energies= -538.794865

8	1	0	3.360343	0.000000	1.968786
9	1	0	3.397631	0.000000	-0.501805
10	6	0	-1.214825	0.000000	-0.698949
11	6	0	-1.230927	0.000000	-2.099677
12	6	0	0.019346	0.000000	-2.832869
13	6	0	1.232894	0.000000	-2.123342
14	6	0	1.250218	0.000000	-0.717701
15	1	0	-2.152160	0.000000	-0.153488
16	6	0	-2.479252	0.000000	-2.829823
17	1	0	2.170377	0.000000	-2.668887
18	6	0	-2.487714	0.000000	-4.201806
19	1	0	-3.409541	0.000000	-2.273141
20	1	0	-3.424135	0.000000	-4.745949
21	6	0	-1.266508	0.000000	-4.910337
22	6	0	-0.026716	0.000000	-4.231620
23	1	0	-1.273850	0.000000	-5.994748
24	1	0	0.892581	0.000000	-4.806558

$C_{14}H_{10}^{2+}$: S_2
Charge = 2 Multiplicity = 1

(E_{rel} = 2.35 eV)

#B3LYP/6-311+G(2d,p) TD(NStates=1, Root=2) opt freq

Low frequencies --- -6.3768 -3.0319 -2.1604 -0.0008 -0.0008 -0.0005
Low frequencies --- 82.0603 104.6265 206.9561

Zero-point correction= 0.190926 (Hartree/Particle)
Thermal correction to Energy= 0.201091
Thermal correction to Enthalpy= 0.202035
Thermal correction to Gibbs Free Energy= 0.155435
Sum of electronic and zero-point Energies= -538.705363
Sum of electronic and thermal Energies= -538.695198
Sum of electronic and thermal Enthalpies= -538.694253
Sum of electronic and thermal Free Energies= -538.740853

Center Number	Atomic Number	Atomic Type	Coordinates (Angstroms) X	Y	Z
1	6	0	-0.715442	1.232205	0.000000
2	6	0	-1.402046	2.476935	0.000000
3	6	0	-0.695835	3.692524	0.000000
4	6	0	0.695835	3.692524	0.000000
5	6	0	1.402046	2.476935	0.000000
6	1	0	-2.486983	2.486679	0.000000
7	1	0	-1.243799	4.627079	0.000000
8	1	0	1.243799	4.627080	0.000000
9	1	0	2.486983	2.486679	0.000000
10	6	0	-1.407390	0.000000	0.000000
11	6	0	-0.715442	-1.232205	0.000000
12	6	0	0.715442	-1.232205	0.000000
13	6	0	1.407390	0.000000	0.000000
14	6	0	0.715442	1.232205	0.000000
15	1	0	-2.492391	0.000000	0.000000
16	6	0	-1.402046	-2.476935	0.000000
17	1	0	2.492391	0.000000	0.000000
18	6	0	-0.695835	-3.692524	0.000000
19	1	0	-2.486983	-2.486679	0.000000
20	1	0	-1.243799	-4.627079	0.000000
21	6	0	0.695835	-3.692524	0.000000
22	6	0	1.402046	-2.476935	0.000000
23	1	0	1.243799	-4.627079	0.000000
24	1	0	2.486983	-2.486679	0.000000

Vibronic calculations:
Energy = 0.0000 cm^-1: |0> -> |0>
-> Intensity = 0.2896E+08 (DipStr = 2.337)

Energy = 388.0088 cm^-1: |0> -> |8^1>
-> Intensity = 0.8144E+07 (DipStr = 0.6430)

Energy = 776.0175 cm^-1: |0> -> |8^2>
-> Intensity = 0.1100E+07 (DipStr = 0.8501E-01)

Energy = 1256.7186 cm^-1: |0> -> |42^1>
-> Intensity = 0.7387E+06 (DipStr = 0.5564E-01)

Energy = 1404.5440 cm^-1: |0> -> |46^1>
-> Intensity = 0.3186E+07 (DipStr = 0.2381)

Energy = 1792.5527 cm^-1: |0> -> |46^1;8^1>
-> Intensity = 0.8564E+06 (DipStr = 0.6273E-01)

$C_{14}H_{10}^{2+}$: S_3
Charge = 2 Multiplicity = 1

(E_{rel} = 3.03 eV)

Low frequencies --- -3.6835 -3.6507 -1.9672 -0.0006 0.0001 0.0003
Low frequencies --- 23.5264 105.7827 144.9559

Zero-point correction= 0.185266 (Hartree/Particle)
Thermal correction to Energy= 0.195755
Thermal correction to Enthalpy= 0.196700
Thermal correction to Gibbs Free Energy= 0.148452
Sum of electronic and zero-point Energies= -538.680326
Sum of electronic and thermal Energies= -538.669837
Sum of electronic and thermal Enthalpies= -538.668893

Center Number	Atomic Number	Atomic Type	Coordinates (Angstroms) X	Y	Z
1	6	0	0.000000	0.000000	0.000000
2	6	0	0.000000	0.000000	1.414923
3	6	0	1.167229	0.000000	2.158415
4	6	0	2.409592	0.072357	1.507893
5	6	0	2.461324	0.091739	0.126584
6	1	0	-0.960899	0.069310	1.924298
7	1	0	1.126125	-0.037210	3.241265
8	1	0	3.323729	0.088019	2.090616
9	1	0	3.425923	0.037867	-0.376605
10	6	0	-1.143686	0.040078	-0.805274
11	6	0	-1.156396	0.027892	-2.204470
12	6	0	0.135831	0.130186	-2.883272
13	6	0	1.276840	0.077602	-2.074529
14	6	0	1.294077	0.102234	-0.675321
15	1	0	-2.100104	0.210237	-0.301395
16	6	0	-2.320379	0.055900	-3.008483
17	1	0	2.227539	-0.136192	-2.575951
18	6	0	-2.268431	0.082794	-4.391159
19	1	0	-3.284929	0.125257	-2.506075
20	1	0	-3.182973	0.066549	-4.973383

Sum of electronic and thermal Free Energies= -538.717140<br><br>Vibronic calculations:<br>Energy = 0.0000 cm^-1: |0> -> |0><br>-> Intensity = 12.87 (DipStr = 0.1508E-04)	21 6 0 -1.026293 0.155257 -5.042054<br>22 6 0 0.139669 0.147818 -4.299330<br>23 1 0 -0.985799 0.192018 -6.124858<br>24 1 0 1.101399 0.094134 -4.807979<br>---

$C_{14}H_{10}^{+}$: D_0

(E_{rel} = -11.71 eV)

Charge = 1 Multiplicity = 2

Low frequencies --- -0.0007 -0.0005 -0.0004 2.6439 3.6320 5.4001
Low frequencies --- 85.0196 118.9532 218.2596

Zero-point correction= 0.193669 (Hartree/Particle)
Thermal correction to Energy= 0.203240
Thermal correction to Enthalpy= 0.204184
Thermal correction to Gibbs Free Energy= 0.158068
Sum of electronic and zero-point Energies= -539.222021
Sum of electronic and thermal Energies= -539.212450
Sum of electronic and thermal Enthalpies= -539.211506
Sum of electronic and thermal Free Energies= -539.257622

Center Number	Atomic Number	Atomic Type	Coordinates (Angstroms) X	Y	Z
1	6	0	-0.014787	0.000000	0.023792
2	6	0	0.013443	0.000000	1.433706
3	6	0	1.221353	0.000000	2.113019
4	6	0	2.432193	0.000000	1.408404
5	6	0	2.438361	0.000000	0.022593
6	1	0	-0.920008	0.000000	1.983919
7	1	0	1.229921	0.000000	3.195614
8	1	0	3.369197	0.000000	1.950723
9	1	0	3.377913	0.000000	-0.517136
10	6	0	-1.223201	0.000000	-0.694081
11	6	0	-1.250306	0.000000	-2.099382
12	6	0	-0.009028	0.000000	-2.821708
13	6	0	1.199385	0.000000	-2.103837
14	6	0	1.226492	0.000000	-0.698534
15	1	0	-2.160765	0.000000	-0.148490
16	6	0	-2.462177	0.000000	-2.820509
17	1	0	2.136951	0.000000	-2.649424
18	6	0	-2.456009	0.000000	-4.206320
19	1	0	-3.401728	0.000000	-2.280780
20	1	0	-3.393013	0.000000	-4.748639
21	6	0	-1.245169	0.000000	-4.910936
22	6	0	-0.037259	0.000000	-4.231625
23	1	0	-1.253739	0.000000	-5.993532
24	1	0	0.896192	0.000000	-4.781836
